# Monitoring SARS-CoV-2 variants in wastewater of Dhaka City, Bangladesh: approach to complement public health surveillance systems

**DOI:** 10.1186/s40246-023-00505-4

**Published:** 2023-07-07

**Authors:** Rehnuma Haque, Mohammad Enayet Hossain, Mojnu Miah, Mahbubur Rahman, Nuhu Amin, Ziaur Rahman, Md. Shariful Islam, Mohammed Ziaur Rahman

**Affiliations:** 1 Environmental Interventions Unit, Infectious Diseases Division, icddr,b, 68 Shaheed Tajuddin Ahmed Sarani, Mohakhali, Dhaka, 1212 Bangladesh; 2 Department of Women’s and Children’s Health, Uppsala University, Akademiska Sjukhuset, 75185 Uppsala, Sweden; 3 One Health Laboratory, Infectious Diseases Division, icddr,b, 68 Shaheed Tajuddin Ahmed Sarani, Mohakhali, Dhaka, 1212 Bangladesh; 4 Institute for Sustainable Futures, The University of Technology Sydney, 235 Jones St, Ultimo, NSW 2007 Australia; 5 The School of Public Health, The University of Queensland, Brisbane, QLD 4072 Australia

**Keywords:** COVID-19, Variants, Wastewater, Lineages, Sequencing, Low-middle-income countries

## Abstract

**Background:**

Wastewater-based epidemiological surveillance has been considered a powerful tool for early detection and monitoring of the dynamics of SARS-CoV-2 and its lineages circulating in a community. This study is aimed to investigate the complexity of SARS-CoV-2 infection dynamics in Dhaka city by examining its genetic variants in wastewater. Also, the study seeks to determine a connection between the SARS-CoV-2 variations detected in clinical testing and those found in wastewater samples.

**Results:**

Out of 504 samples tested in RT-qPCR, 185 (36.7%) tested positive for SARS-CoV-2 viral RNA. The median log_10_ concentration of SARS-CoV-2 N gene copies/Liter of wastewater (gc/L) was 5.2, and the median log_10_ concentration of ORF1ab was 4.9. To further reveal the genetic diversity of SARS-CoV-2, ten samples with ORF1ab real-time RT-PCR cycle threshold (Ct) values ranging from 28.78 to 32.13 were subjected to whole genome sequencing using nanopore technology. According to clade classification, sequences from wastewater samples were grouped into 4 clades: 20A, 20B, 21A, 21J, and the Pango lineage, B.1, B.1.1, B.1.1.25, and B.1.617.2, with coverage ranging from 94.2 to 99.8%. Of them, 70% belonged to clade 20B, followed by 10% to clade 20A, 21A, and 21J. Lineage B.1.1.25 was predominant in Bangladesh and phylogenetically related to the sequences from India, the USA, Canada, the UK, and Italy. The Delta variant (B.1.617.2) was first identified in clinical samples at the beginning of May 2021. In contrast, we found that it was circulating in the community and was detected in wastewater in September 2020.

**Conclusion:**

Environmental surveillance is useful for monitoring temporal and spatial trends of existing and emerging infectious diseases and supports evidence-based public health measures. The findings of this study supported the use of wastewater-based epidemiology and provided the baseline data for the dynamics of SARS-CoV-2 variants in the wastewater environment in Dhaka, Bangladesh.

**Supplementary Information:**

The online version contains supplementary material available at 10.1186/s40246-023-00505-4.

## Background

SARS-CoV-2 is primarily detected in the respiratory tract; however, it has also been found in the gastrointestinal tract, where it persists for a long time. SARS-CoV-2 viral shedding starts in the urine and faeces immediately; the patient is infected with SARS-CoV-2 even if they do not have typical symptoms like fever, cough, or breathing difficulties [1, 2]. As a result, monitoring wastewater has been used to isolate and quantify SARS-CoV-2 viral RNA in sewage in many countries worldwide since the COVID-19 pandemic, with a strong association between viral RNA genomic copies and the number of reported cases of COVID-19 [3–8]. In addition to the viral quantification in wastewater, genomic analysis is a promising tool for investigating the spread of SARS-CoV-2 variants circulating in the community via real-time quantitative PCR (RT-qPCR) [9–14].

Throughout the pandemic, several rapidly spreading SARS-CoV-2 lineages have been identified globally. The reported lineages are termed variants of concern (VOCs) depending on their nature of infectivity and transmissibility and first-time reporting from countries such as the United Kingdom (UK), South Africa (SA), Brazil (BZ), India, and the United States of America (USA). These VOCs include Alpha (B.1.1.7), Beta (B.1.351), Gamma (P.1), Delta/Kappa (B.1.617), and, more recently, the C.1.2 lineage [15, 16]. These mutations occur due to the multiple changeovers in the viral spike (S) glycoprotein [17]. In this study, attempts were made to retrieve whole SARS-CoV-2 genome sequences from RNA quantified in wastewater samples across different sanitation points of Dhaka city from September 2020 to February 2021 to determine the frequencies of VOCs and the diversity of the infection dynamics present in the adjacent community. Wastewater treatment is almost absent in Dhaka city. Only 20% of sewage undergoes treatment. Even the on-site sanitation system does not function properly because of a lack of strong monitoring. Less than 3.0% of domestic sewage is treated, and most people discharge untreated wastewater down drains into adjacent water bodies or rivers [18]. Due to the significantly low sanitation coverage and the lack of sewage treatment plants in Bangladesh, it is highly likely that SARS-CoV-2 may be present in the sewage in Dhaka. The lack of adequate wastewater treatment infrastructure is a major concern, as it increases the risk of enteric pathogen transmission through environmental exposure routes. In addition, the absence of proper sanitation facilities poses a significant public health risk, particularly in densely populated urban areas where wastewater is often discharged into open drains and waterways. This can lead to the contamination of water sources and exposure to harmful pathogens, including SARS-CoV-2. There is a theory that fecal-airway transmission of SARS-CoV-2 may occur through the inhalation of fecal particles that contain viable viruses in the form of aerosol droplets [19, 20]. The theory is based on findings that SARS-CoV-2 can be detected in fecal samples, suggesting that the virus can be shed through the gastrointestinal tract. If viable SARS-CoV-2 virus is present in fecal matter, it could potentially become aerosolized and spread through the air, leading to the potential transmission through inhalation. A recent systematic review found supporting evidence from two studies showing successful infection in 56.5% of cases, suggesting the possibility of fecal–oral transmission of SARS-CoV-2 [20]. However, this is still speculative and requires further confirmation through rigorous scientific studies. A recent study conducted in Mexico has reported the presence of live SARS-CoV-2 virus in municipal wastewater. The findings indicate a statistically significant, albeit low, correlation between the percentage of treated wastewater and the number of positive coronavirus cases. However, caution should be exercised in interpreting these results, as wastewater is not considered a primary transmission mechanism for the virus [21]. As per the World Health Organization (WHO), there is currently no evidence to suggest that the COVID-19 virus can be transmitted through sewerage systems, with or without wastewater treatment [22].

Therefore, the successful identification of SARS-CoV-2 variants in diverse sanitation systems, including treated and untreated sewage, demonstrates the importance of wastewater monitoring, a robust surveillance tool for early infection detection and preparedness of public health authorities [23]. This pilot research aims to explore SARS-CoV-2 infection dynamics in Dhaka city by conducting wastewater monitoring. This research also aims to establish a correlation between the SARS-CoV-2 variants identified in clinical testing and those detected in wastewater samples. Using this approach, the pilot study aims to provide valuable insights into the spread and evolution of the variants, which could inform public health strategies to control and mitigate the COVID-19 pandemic.

## Methods

### Sampling points

In this study, we have collected grab samples repeatedly every week from different 09 sampling sites. These locations included different points of the community drains and canals, hospital septic tanks, pumping stations, and a wastewater treatment plant (WWTP) from August 2020 to May 2021. The clinical case data were retrieved from MIS/DGHS (www.mis.gov). Bangladesh government data at the same period found a similar trend. We scheduled our sampling time in the morning, as people typically use the toilet before leaving their homes during that time. In our research protocol, we established a maximum period of sample transportation of 6 h. However, the majority of the samples were transported to the laboratory within 2 h, usually before 10 am. We strictly adhered to the guidelines provided by the Centers for Disease Control and Prevention (CDC) to avoid any pre and post-exposure with sewage samples [24]. To ensure the identification of the wastewater flow from upstream to downstream, we carefully selected the sampling points. Our hypothesis was that the downstream samples would contain more viral gene copies, as these samples represent the mixing of different wastewater sources. The grab sampling method was chosen because it is a quick and easy way to collect wastewater samples in Bangladesh. We ensured proper labeling of the samples to avoid cross-contamination or any other issue that could affect the integrity of the results. This approach allowed us to obtain high-quality samples that were representative of the different wastewater sources in the community. We collected samples from the influent and effluent of the wastewater treatment plant (WWTP). The WWTP performed biological treatment before releasing it into the environment. We chose to sample both points to get a comprehensive understanding of the efficiency of the treatment process in removing SARS-CoV-2 from the wastewater. The selection of these sampling points allowed us to capture the presence of SARS-CoV-2 in different stages of the wastewater treatment process. Additionally, we collected samples from the inlets of fourteen active pumping stations that transport sewage water from the community to the WWTP. The comprehensive sampling strategy provided us with a detailed understanding of the distribution and persistence of SARS-CoV-2 in the wastewater system in Dhaka city. These facilities were listed from the Dhaka Water Supply and Sewerage Authority.

Commonly, septic tanks are categorized into two types, single-chambered septic tanks and multiple-chambered septic tanks (ABR). A single-chambered septic tank is confined to one chamber with one or more openings on the top and no outlet on its sidewall, and the sample was collected from this chamber. ABRs have two or more chambers with an inlet in the first chamber and an outlet in the last chamber. ABRs are functional only when each of the chambers is sealed with a lid. The sample was collected from the first chamber of the ABR, to which the inlet was connected.

Next, we carried out wastewater sampling from different tiers of drains within a defined catchment area in the community. The catchment area was selected to represent a specific community or residential area with a distinct set of sewerage and drainage infrastructure. We classified the drains into three tiers based on their location and characteristics. For example, a third-tier drain refers to any sewage pipe originating from a community's residential dwelling. These pipes can be either open-ended or inlet pipes that lead to a household septic tank. For sampling purposes, the collection point is situated close to the household septic tank. A second-tier drain is an underground sewage system under community roads. We took samples at the meeting points of branch roads that connect to the main road. We also took samples from the endpoint of the community drain to represent all the wastewater that flows into bodies of water. We chose these sampling points to obtain representative samples from all sources of wastewater in the community.

### Collection method

Our field team waded into the water to avoid debris and utilized a large metal ladle to gather 400 mL of drain water. A 500 mL clean mug was positioned at the sampling point to collect the drain water. Once collected, the samples were transferred into a 2 L Whirl–Pak bag for further processing. To ensure the preservation of the samples, the field assistant immediately placed them into a cold box that was maintained at a temperature below 10 °C with the aid of ice packs. Within six hours, the samples were transported to the icddr,b laboratory for testing. Upon arrival, the laboratory staff received the samples and placed them in a refrigerator set at 4 °C to maintain their integrity until testing.

### Sample processing

**Fig. 1:**
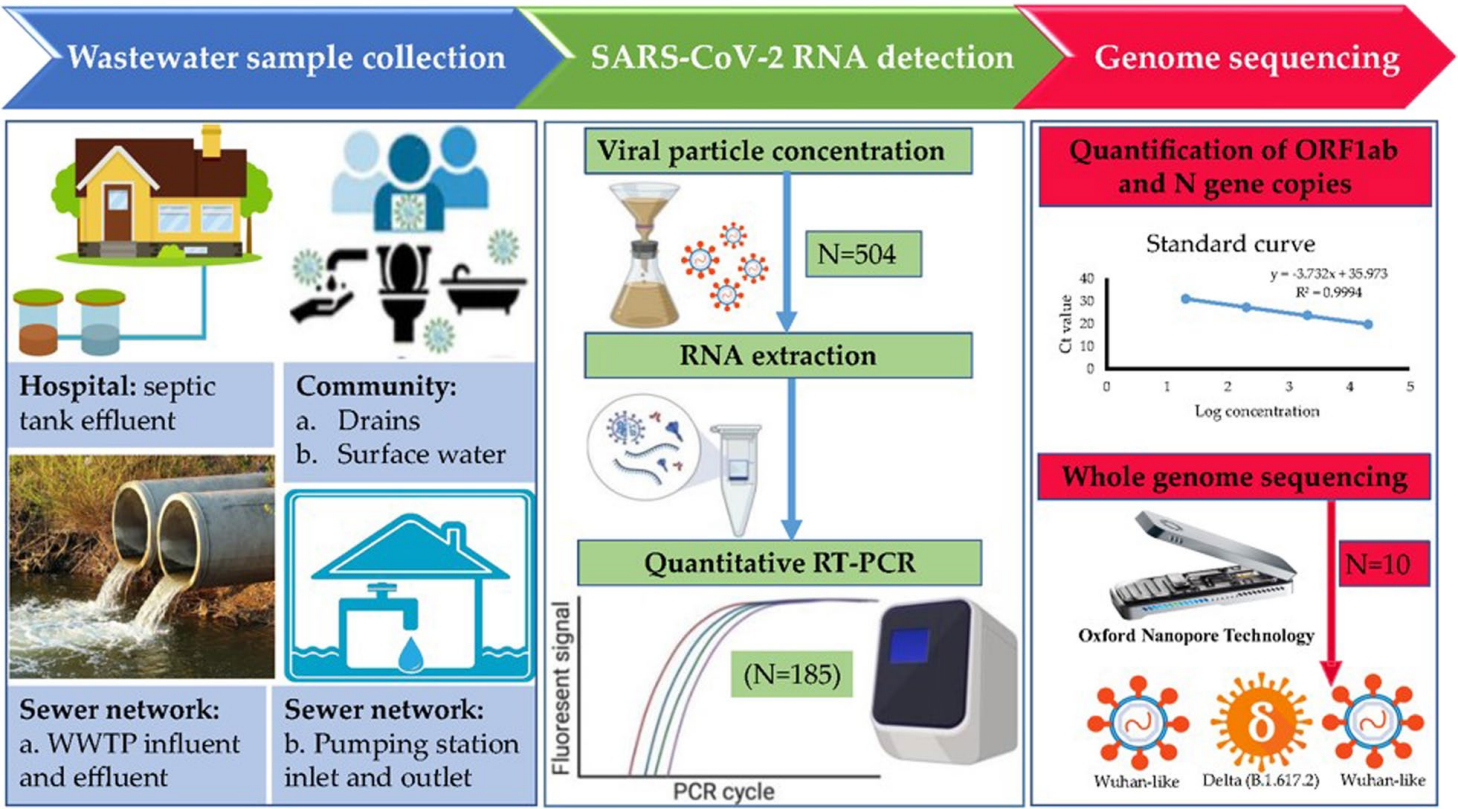
A schematic diagram describing the integrative process of sample collection and laboratory analysis

SARS-CoV-2 viruses were concentrated using a modified calcium flocculation-citrate dissolution method described elsewhere [25]. The overall workflow from the sample collection to variant detection is illustrated in Fig. 1. To an approximate 20–200 mL water sample, 0.1–0.4 mL 1 M CaCl_2_ and 0.1–0.4 mL 1 M Na_2_ HPO_4_ were added, followed by stirring the mixture for 5 min in order to form flocculation. Subsequently, a non-charged hybrid cellulose ester membrane (pore size 0.45 m and diameter 50 mm) was then used to filter the solution using a Millipore manifold filtration system (EZ-Fit™ Manifold, Merck KGaA, Darmstadt, Germany). After filtration, the membrane was transferred into a sterile 3.5 mL storage vial for further processing to extract viral RNA. In the viral RNA concentration process, we used a modified calcium flocculation-citrate dissolution method. However, we did not use trisodium citrate dissolution as we used a half portion of the filter membrane directly and a bead beating method to dissolve viral RNA into a lysis solution. The lysis solution containing viral genetic material was used to extract the RNA. Since wastewater contains contaminants (humic acids and other inhibitors of PCR), it is critical to remove them as they might interrupt PCR amplification. Therefore, we used the Norgen Soil Total RNA Purification Kit, which yields high-quality total RNA and removes humic acids and other PCR inhibitors.

### Viral RNA extraction and SARS-CoV-2 RNA detection

All the environmental samples were processed at the One Health Laboratory, icddr,b. Using autoclaved scissors and forceps, a half-portion of the filter membrane (approximately 300 mg) containing the concentrated environmental sample was cut and dissolved in 700 µL of Lysis Buffer I, followed by bead mixing for 5 min using a bead beater at maximum speed. Viral RNA was extracted from the processed samples using the Norgen Soil Total RNA Purification Kit (Norgen Biotek Corporation, Ontario, Canada). A mock blank control was used in each batch of sample processing to check contamination during sample processing and RNA extraction. One-step RT-qPCR was performed for the detection of SARS-CoV-2 viral RNA targeting ORF1ab and N genes using the iTaq Universal Probes One-Step kit (Bio-Rad Laboratories, CA, USA) following the manufacturer’s instructions in a duplex manner [26]. Real-time RT-qPCR was carried out in a final reaction volume of 25 µL containing 1 × iTaq universal probe reaction mix, 0.5 µL of RT enzyme, 400 nM of each forward and reverse primer, 200 nM of each probe, 5 µL of template RNA/control, and molecular grade water to equilibrium the final volume. The thermal cycling conditions were reverse transcribed for 10 min at 50 °C, 3 min at 95 °C, 45 cycles for 15 s at 95 °C, and 45 s at 60 °C. The PCR reaction was carried out in the CFX Opus 96 Real-Time PCR System (Bio-Rad Laboratories, CA, USA). The presence of viral RNA was detected if either one or both of the primer and probe sets were amplified. The wastewater samples were categorized as ‘positive’ for SARS-CoV-2 viral RNA when at least one target was amplified with Ct values ≤ 38.

### Viral load estimation using qPCR

To construct a standard curve for the quantification of SARS-CoV-2 viral RNA load in the wastewater, we ordered a plasmid construct to insert our desired sequence from GenScript (GenScript USA Inc., Piscataway, NJ, USA). First, lyophilized plasmid (4 µg) was dissolved in 100 µL of TE buffer to obtain the stock plasmid concentration of 40 ng/µL. Next, this reconstituted stock was diluted 100 times to yield a concentration of 0.4 ng/µL (1.07 × 10^8^ copies/µL) since the plasmid construct was 3470 bp in size. Later, a set of qPCR standards were prepared by tenfold serial dilutions with concentrations starting from 1.07 × 10^7^ copies/µL to 1.07 × 10^4^ copies/μL. At every PCR setup, two no-template controls (NTCs) were used to monitor the master mix contamination, and two standards for each dilution were used to generate the standard curve.

Standard curve calculations were based on standard dilution series ranging from 2.14 × 10^3^ to 2.14 × 10^6^ copies per μL of the PCR reaction. From each RT-qPCR run with serially diluted standards, we constructed a standard curve based on the log copy number of each standard dilution and obtained the average Ct value of the particular standard dilution. From the standard curve, we obtained the linear regression value (R^2^) and the slope-intercept form of the equation of the straight line (*y* = *mx* + *c*), where m is the gradient of the line (how steep the line is), and c is the y-intercept. Using the intercept and slope value, we calculated the input RNA copy number per microliter template from the equation, Nn = 10 ^((Ct−C)/m)^, where Nn is the input template copy number. After obtaining the RNA copy number per µL of reaction volume and input template, we back-calculated the viral RNA copy numbers per ml of a sample using the formula copy/mL = (copies/µL × total elute)/sample (mL). Initially, the gene copy number was quantified per μL of sample input RNA as described above, and the back-calculation was performed based on the initial gene copy number, total eluted RNA, and total concentrate, extracted concentrate of wastewater.

### SARS-CoV-2 genome sequencing and analysis

Among the 504 tested wastewater samples, thirty had Ct values ≤ 32.9. We randomly selected every third of positive samples (*n* = 10) for whole genome sequencing (Ct value range 28.78–32.13) to get an overview of variant circulation in the community. Oxford Nanopore sequencing library was prepared following the ARTIC nCoV-2019 sequencing protocol v3 (LoCost) with some modifications [27, 28]. Briefly, ten SARS-CoV-2 viral RNA was reverse transcribed, and the second strand was synthesized in two pools. Amplified PCR products were pooled and diluted based on the quantification report. Diluted amplicons were subjected to an end-prep reaction and barcoded by native barcode expansion packs EXP-NBD104. Barcoded amplicons were pooled, and a 0.4 × AMPure XP bead (Beckman Coulter, California, USA) purification was carried out following the protocol. The library was quantified by the Qubit 1 × dsDNA High Sensitivity Assay Kit (Invitrogen, Oregon, USA) with a fluorometer (Qubit 4; Invitrogen, Oregon, USA). The purified barcoded amplicon pool was taken forward to the sequencing adapter ligation step with ONT Adapter Mix II (AMII). Final libraries were quantified, and approximately 38 ng were loaded on the FLO-MIN106D flow cell on an Oxford Nanopore MinION MK 1C platform for 10 h. Real-time base-calling and barcode demultiplexing were carried out with Guppy 4.3.4, released with MinKNOW software v21.06 with the fast base-calling mode. QC passed fastq reads were trimmed by Porechop v.0.2.3. Subsequently, reference-based assembly was performed using pairwise aligner minimap2 with the “-ax map-ont” setting [29]. Mapped files were inspected in the Integrative Genomics Viewer (IGV) v.2.12.2, and the quality and map coverage of the alignments were checked by qualimap tool v.2.2.2. Variant calling under 10 × read depth was filtered**,** and consensus fastq was generated by SAMtools and BCFtools (v 1.5.0) via the mpileup command [30]**.** Finally, consensus FASTA files were generated from consensus fastq via the seqtk tool. The quality of consensus fasta was checked, and a phylogenetic tree was constructed using the Nextclade v1.5.2 (https://clades.nextstrain.org/) with default parameters.

## Results

**Fig. 2:**
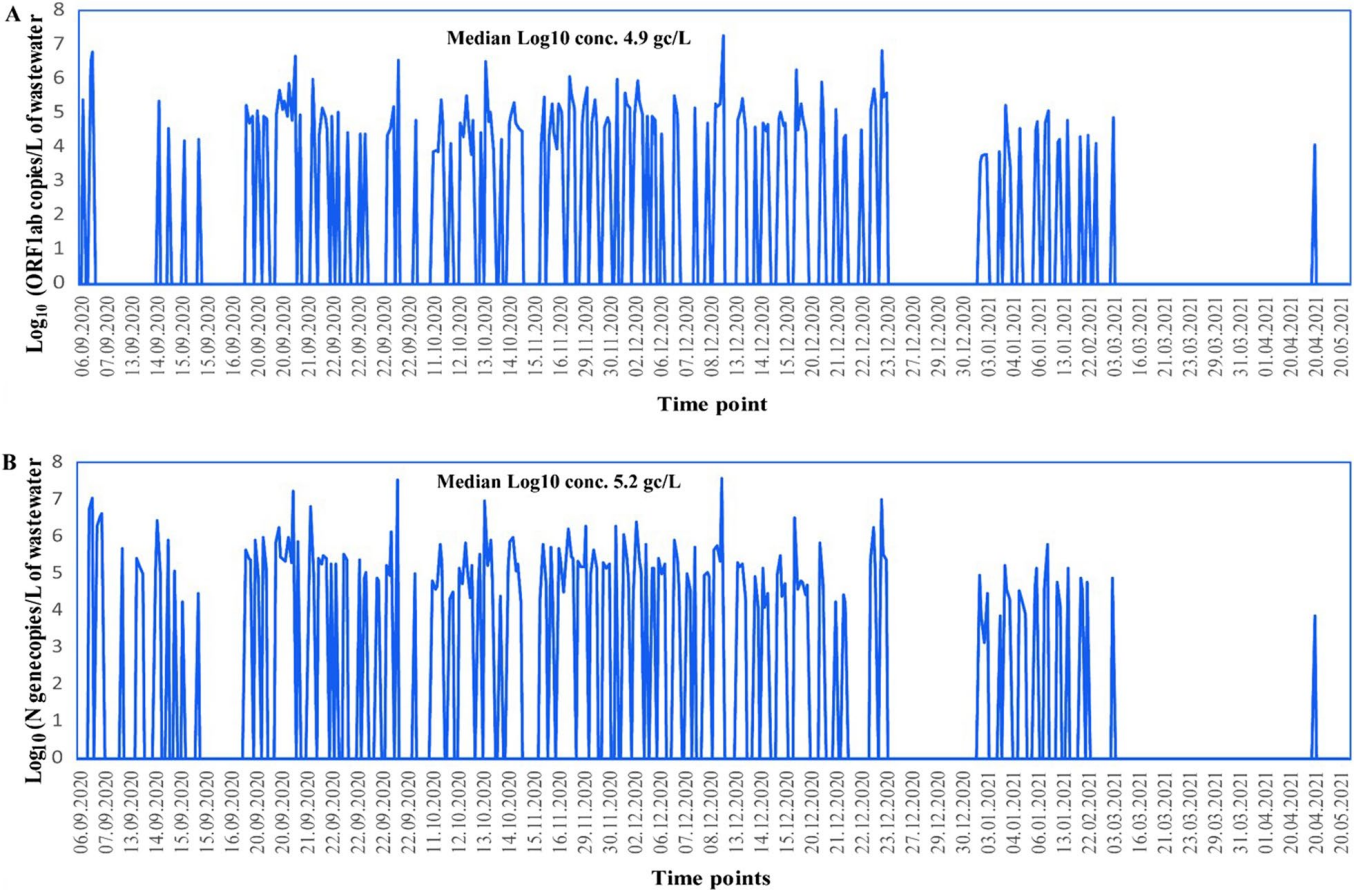
The Log_10_ SARS-CoV-2 ORF1ab and N gene concentration measured in wastewater samples from environmental surveillance in Dhaka city from Sep 2020 to May 2021. The concentration was measured in per liter of wastewater sample. The Median Log_10_ concentration for ORF1ab was 4.9 gc/L (**A**) and for N gene 5.2 gc/L (**B**)

Water samples collected from different locations in Dhaka City were concentrated by the calcium flocculation method, and the levels of SARS-CoV-2 viral RNA were investigated by one-step RT-qPCR. The studied samples were collected in a time frame of 257 days (6 September 2020–20 May 2021). Of the 504 samples collected, 185 (36.7%) were positive for SARS-CoV-2 (Additional file 1: Figure S1). As mentioned beforehand, the wastewater samples were categorized as ‘positive’ for SARS-CoV-2 if at least one target was amplified and Ct values ≤ 38. SARS-CoV-2 viral RNA copy numbers were measured in the water specimens. This study calculated the lowest gene copy at 28 copies/mL of environmental samples, at CT values 38 for ORF1ab. The median log_10_ concentration of SARS-CoV-2 N gene copies/Liter of wastewater (gc/L) was 5.2 (range = 3.9–7.6), and for ORF1ab, it was 4.9 (range = 3.96–7.3). There was no significant difference in the viral log_10_ gene concentration during the study period. The strongest coherence between the viral load of the environmental surveillance samples and the number of clinical cases was found during the period. Although Bangladesh faced its first wave of COVID-19 in April 2021, positivity was relatively low in wastewater during that time period compared to increased COVID-19 cases in clinical samples. However, the frequency of sampling was also low (Fig. 2).

**Fig. 3:**
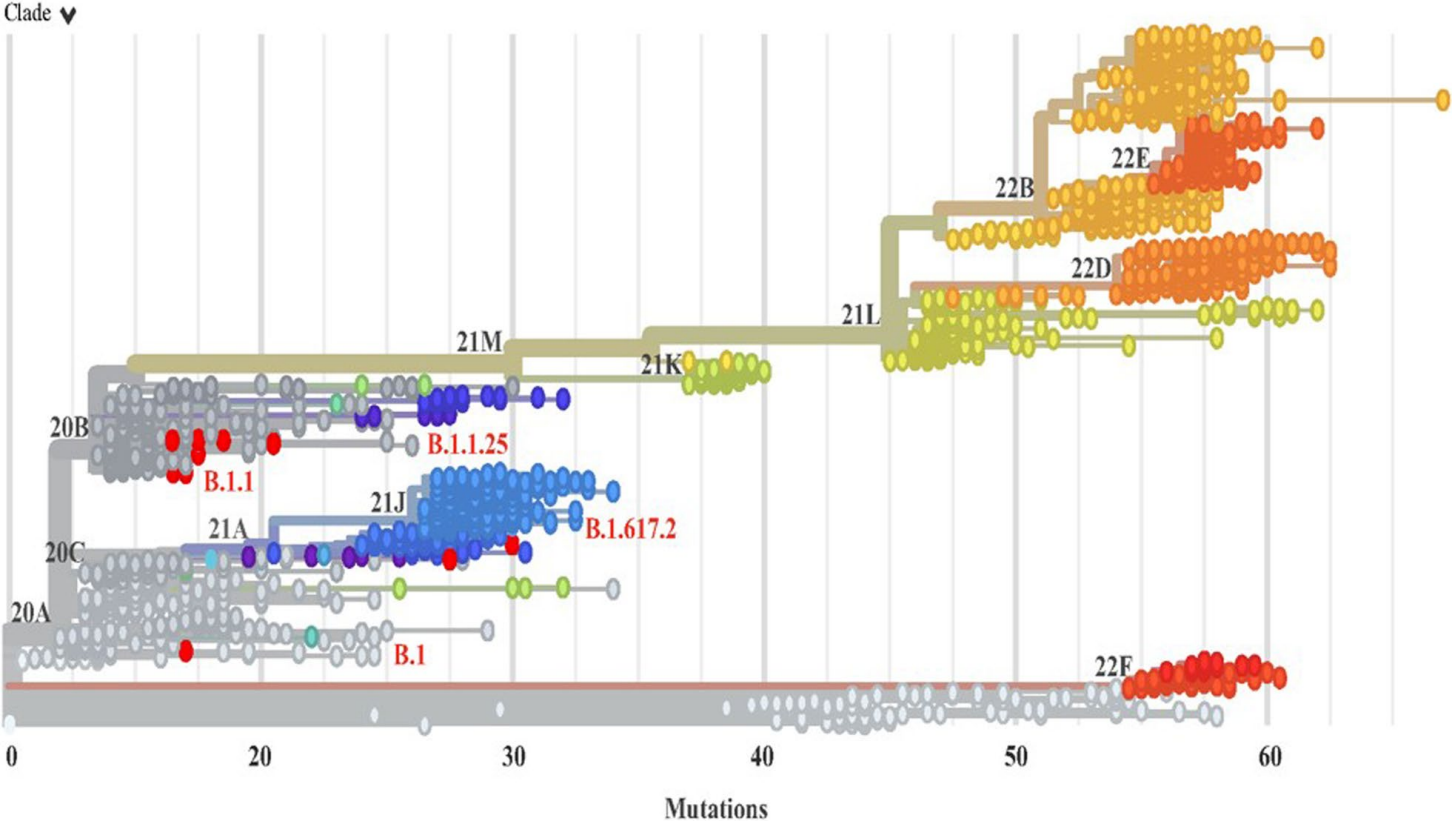
Phylogenetic analysis of SARS-CoV-2 genomes detected in wastewater in Dhaka city Bangladesh. The phylogenetic tree was constructed using the Nextclade v1.5.2 (https://clades.nextstrain.org/) with default parameters. Red colored circular strains are from this study

To further probe the genetic diversity of SARS-CoV-2, ten wastewater samples with ORF1ab cycle threshold (Ct) values ranging from 28.78–32.13 were subjected to whole-genome sequencing using Oxford Nanopore Technology. The genome sequence of all the attempted SARS-CoV-2 positive samples was retrieved successfully with 94.2 to 99.80 genome coverage. According to clade classification using the Nextclade v2.13.0, the sequences from water samples in Dhaka city were grouped into four clades: 19B, 20A, 20B, and 21A (Table 1, Fig. 3), where 70% belonged to clade 20B, followed by 10% to clade 20A, 21A, and 21J. Till the end of 2020, lineage B.1.1.25 was predominant in Bangladesh and phylogenetically related to the sequences from India, the USA, Canada, the UK, and Italy [31]. We also detected lineage B.1.1.25 in 50% of the sequenced samples, which is congruous with the circulation of variants in clinical samples in the country. Strikingly, we detected two COVID-19 Delta variants (B.1.617.2). Although the Delta variant (B.1.617.2) was first reported in clinical samples at the beginning of May 2021 in Dhaka city [32, 33]. In contrast, we found that it was circulating in the community and was detected in wastewater in September 2020. There are possible explanations for these findings of delta variant in wastewater earlier than clinical samples; (1) in Bangladesh, there was no extensive sequencing-based variant surveillance program; (2) although the delta variant was first reported in India. However, the first human case was reported in the USA on 12 march 2020, even though, later, India reported delta variant cases on 1 May 2020 (based on metadata on GISAID), which was too earlier than the first known report on October 2020 in India. Many countries reported human cases infected by delta variants before October 2020, including the USA, Iran, Australia, Senegal, France, Germany, Egypt, and DRC. In addition, the delta variant was also reported in wastewater before October 2020 (Table 2). We also performed mutation analysis in our sequences. Amino acid changes compared to the SARS-CoV-2 reference sequence are listed in Table 3.

**Table 1:** Overview of wastewater samples and variant information of detected SARS-CoV-2

Sample ID	Sampling date	Ct_ORF1ab	Coverage (%)	Nextclade	Pango lineage (nextclade)	Variant label
030106	6 September, 2020	28.78	94.20	20B	B.1.1.25	Wuhan-like variant
0101244	9 December, 2020	29.01	99.80	20B	B.1.1.25	Wuhan-like variant
0101082	21 September, 2020	29.59	97.80	20B	B.1.1.25	Wuhan-like variant
0101142	13 October, 2020	29.65	98.20	20B	B.1.1.25	Wuhan-like variant
030105	6 September, 2020	29.73	97.40	20B	B.1.1	Wuhan-like variant
0101309	23 December, 2020	31.02	98.30	20B	B.1.1	Wuhan-like variant
0401132	22 September, 2020	31.22	96.70	20A	B.1	Wuhan-like variant
202,080	20 September, 2020	31.53	96.50	21J	B.1.617.2	Delta
0102275	16 December, 2020	31.66	99.00	20B	B.1.1.25	Wuhan-like variant
0102089	21 September, 2020	32.13	94.20	21A	B.1.617.2	Delta

**Table 2:** Detection of SARS-CoV-2 variants in wastewater surveillance

First reported in human	First outbreak	First human case*	First report in WW*	Variant	WHO label	Transmissibility	Wastewater sample type
May 2020	South Africa	January, 2020	April, 2021	B.1.351	Beta	50%	Sewage treatment facilities
September 2020	UK	February, 2020	January, 2021	B.1.1.7	Alpha	43–90%	Sewage
October 2020	India			B.1.617.1	Kappa	–	WWTP
October 2020	India	March, 2020	September, 2020	B.1.617.2	Delta	–	WWTP [53]
November 2020	USA			B.1.526	Iota	20%	WWTP
November 2020	Brazil	April, 2020	May, 2021	P. 1	Gamma	152%	WWTP
November 2021	Botswana	November, 2021	November, 2021	B.1.1.529	Omicron	–	Wastewater (Florida)
May 2021 (Bangladesh)	–	–	–	B.1.617.2	Delta	–	–
This study (in wastewater sample)	–	–	–	B.1 B.1.1 B.1.1.25 B.1.617.2	Delta	–	Septic tanks Drains Surface water Pumping station

*WW* Wastewater

*First human case is based on the sequence deposited on GISAID database

**Table 3:** Mutation analysis in sequences detected in our wastewater surveillance study

Sample ID	GISAID Accession number	Genome size (bp)	Amino acid mutations
030106	EPI_ISL_17398164	29,847	NSP2:I120F; NSP3:A1766V, P1228L, P77L; NSP4:A446V; NSP12:P323L; Spike:D614G; N:R203K, G204R
0101244	EPI_ISL_17398165	29,851	NSP2:I120F; NSP3:A1941T; NSP4:A446V, NSP12:P323L; NSP15:D128E; NSP16:T117I; Spike:D614G; T676I; NS3:W193R; N:R203K G204R
0101082	EPI_ISL_17398166	29,839	NSP2:I120F, W450C; NSP3:S1296F; NSP12:P323L NSP13:E261D; Spike:D614G, Q677H, T719I; N:R203K, G204R
0101142	EPI_ISL_17398167	29,878	NSP2:I120F; NS3:T223I; NSP6:V149F; NSP12:P323L; NSP15:T33I M:A142V; Spike:D614G; N:R203K, G204R
030105	EPI_ISL_17398168	29,865	NSP2:P129L; NSP3:P822L; NSP4:A446V; NSP12:P323L Spike:D614G; M:I82T; N:R203K G204R
0101309	EPI_ISL_17398169	29,878	NSP2:P129L; NS3:S26L; NSP4:A446V; NSP12:P323L; Spike:D614G, P681R; N:R203K, G204R
0401132	EPI_ISL_17398170	29,838	NSP3:M1547I; NSP4:A446V; NSP5:P99L; NSP6:T77A; NSP12:P323L, G671S; Spike:D614G; N:R203M
202,080	EPI_ISL_17398171	29,771	NSP3:P1228L, A488S, V1234L, P1469S; NSP4:V167L, T492I; NSP6:A76V, T77A; NS7b:T40I; NSP8:P183L NSP12:P323L, G671S; NSP13:P77L; NSP14:A394V Spike: T19R, E156G, R158del, F157del, G142D, D614G, P681R; M:I82T; N:G215C R203M, D377Y
0102275	EPI_ISL_17398172	29,845	NSP1:R24C; NSP2:I120F; Spike:T19R, A222S, D614G, P681R, A924S; N:R203K G204R
0102089	EPI_ISL_17398173	29,771	NSP3: S26L, H682Y; NSP4:T492I; NSP6:V149A; NSP12: T51I P323L G671S; NSP13:P77L, V558L; NSP15:H234Y; Spike: T19R, E156G, G142D, F157del, R158del, D614G, P681R; M:I82T; N:G215C, R203M

## Discussion

SARS-CoV-2 environmental surveillance in wastewater has several epidemiological implications. Monitoring SARS-CoV-2, for example, can supplement epidemiological data and complement clinical information. Our findings demonstrate that methodologies for quantifying and sequencing SARS-CoV-2 viral RNA in wastewater samples are consistent with clinical sequencing and applicable to relevant variant abundance estimations. Despite the presence of inhibitors, xenobiotics, and diluted copy numbers in the environmental samples, the detection of SARS-CoV-2 RNA was successful, with good genomic coverage of up to 99.8%. We could retrieve 94–99% coverage, although that was fair enough to support the requirement to reveal the viral clade, variant, and lineage information in Nextclade and Pangolin. Since wastewater may contain fragmented RNA, which might affect genome coverage. However, the recovery of > 94% genome coverage from wastewater indicates the efficiency of the study.

As part of the countrywide COVID-19 laboratory network, the International Centre for Diarrhoeal Disease Research, Bangladesh (icddr,b), in collaboration with the Government of Bangladesh, has been testing for SARS-CoV-2 since March 2020 [32]. In Bangladesh, Wuhan-like SARS-CoV-2 variant B.1.1.25 was dominant in clinical samples during December 2020. icddr,b colleagues found a high prevalence of the B.1.1.25 (Wuhan-like) lineage in clinical samples during December 2020 in the rural district of Faridpur, Bangladesh [33]. We found B.1.1.25 lineages circulating in wastewater from early September 2020 to late December 2020 in Dhaka, the capital city of Bangladesh. The Delta variant (B.1.617.2) was first identified in clinical samples at the beginning of May 2021 [32]. The USA also detected both Alpha and Delta variants first in wastewater before clinical samples [34], and based on sequence repository GISAID, the USA reported the first case of delta variant in March 2020. Many other countries like Iran, Australia, Senegal, France, Germany, Egypt, and DRC reported delta variants in clinical samples in March, April, and May 2020. According to sequences deposited in the GISAID database, Alpha, Beta, Gamma, and Delta variants were reported at least six months earlier than the first known report (Table 2). A study was conducted as a part of the EU Sewage Sentinel System for SARS-CoV-2 [35], which demonstrated a clear link between clinical and wastewater SARS-CoV-2 mutation profiles associated with the variants of concern at the time, namely B.1.1.7, P.1, B.1.351, and B.1.617.2, present in 20 European nations. Our study’s findings also align with the aforementioned EU report. Additionally, the all-inclusive study suggests the potential use of a uniform protocol for various wastewater matrices across Europe and potentially worldwide to obtain high-coverage next-generation sequencing (NGS) data of SARS-CoV-2. The produced data suggests the potential to achieve over 98% coverage of the SARS-CoV-2 genome in wastewater samples, which may contain a combination of genomic material from multiple SARSCoV-2 variants. These results indicate cryptic transmission of SARS-CoV-2. Several environmental surveillance studies conducted in the U.S., Italy, France, and India, reported the detection of SARS-CoV-2 variants earlier than the detection in clinical samples [34, 36–38]. Karthikeyan et al. identified multiple instances of virus spread not captured by clinical genomic surveillance [34]. A longitudinal study conducted in Bengaluru, India, detected emerging variants of concern up to two months earlier in wastewater samples [38]. Using wastewater-based epidemiology, our finding provides the baseline data/analysis of SARS-CoV-2 variant dynamics in Bangladesh. Identifying infectious pandemic pathogens like SARS-CoV-2 in wastewater can help make timely decisions and take public health actions by providing the earliest possible dates for initiating lockdowns and health resource management in vulnerable areas with high infection potentiality [39]. These results can expedite the outbreak response, help minimize the occurrence of an infection outbreak, and reduce hospital admission by early forecasting of the disease outbreak. Recent publications in Bangladesh have emphasized the significance and implications of wastewater-based epidemiology. These studies highlight that environmental surveillance can provide unbiased insights into the transmission of SARS-CoV-2, especially when clinical surveillance is inconsistent and may not capture the complete picture. This becomes particularly relevant during periods of limited clinical testing, and the findings align with those observed in high-income settings (e.g., [23, 25, 40–42]. It is worth noting that the sample size we utilized for our wastewater-based epidemiological surveillance/monitoring was relatively small, which may limit the representativeness of our findings. Nonetheless, we observed that the dominant variants in circulation were well-represented in our sample, even though we had expected to capture a broader range of variants. Despite this limitation, our results still provide valuable insights into the prevalence of SARS-CoV-2 in the analyzed wastewater samples in LMICs. The complex nature of wastewater samples poses a significant challenge in identifying mutations and variants of the SARS-CoV-2 virus. The composition of the wastewater matrix is intricate, hindering the retrieval of sufficient SARS-CoV-2 RNA and resulting in low genome coverage. The absence of dedicated sewage channels in Dhaka city has resulted in a substantial obstacle when it comes to wastewater management. However, we managed to collect samples from major pumping stations, which eventually carry all the wastewater to the WWTP located 14 km away from the Dhaka city (Additional file 1: Figure S2). The wastewater in the area contains a high concentration of inhibitors, toxic materials, bleaches, and soaps and detergents from washing and bathing activities. This complex mixture of contaminants can potentially influence the eligibility of SARS-CoV-2 genes during sequencing. Moreover, the diverse composition of wastewater among various regions presents a challenge in establishing a uniform approach to sequencing the SARS-CoV-2 virus in wastewater. As a result, the analysis of SARS-CoV-2 in wastewater is a complex and challenging task that requires careful consideration and optimization of sequencing protocols [43].

Our results indicated that a small volume of wastewater sample (approximately 100–400 mL) collected in this study was able to obtain reliable virus recovery via direct extraction of wastewater. In addition, the deployment of the genome sequencing approach [28] has added a significant impact on the study outcome. In our previous study on wastewater, we have shown the benefits of monitoring the circulation of the variants in wastewater to track the spread of variants in a complex sanitation system [23]. This research suggests, in particular, that rather than collecting a large volume of wastewater samples as suggested by many of the environmental surveillance studies [7, 44], which would necessitate a significant amount of labor, equipment, and time from trained field personnel, this method could deter the utilization of already limited resources for critical surveillance tasks, particularly in low-resource areas. As a result, this type of surveillance operation’s cost–benefit ratio should be carefully assessed before it is implemented. It will be preferable to test the under-structures and diverse sanitation network at many points, as we did for B.1.1.7, to get an idea of the local circulation of the different variants and to identify the clusters better. This should help to determine the emergence of variants to the greatest extent to target the control measures.

Moreover, getting a substantial amount of information and an efficient snapshot of the prevalence of the pathogen in a community requires conducting a study on a larger human population, which can augment costs and be cumbersome. However, monitoring wastewater provides an overall depiction of the types and amounts of pathogens circulating in the community; in fact, a single wastewater specimen gives representative data about an entire ward, town, or county, which reduces a great deal of cost. Results from individual testing should be the most accurate indicator of disease transmission and occurrence in the population, but in countries where population density is high, health facilities are overburdened, and the economic condition is poor, the mass monitoring through sewage creates more comprehensive coverage for detecting emerging pathogens [45]. Sewage samples also have many advantages over clinical samples because they are easier to collect, do not require reaching out to patients, raise fewer ethical concerns and sampling biases, and require fewer samples to get a comprehensive picture of viral diversity in a community, including asymptomatic infections [3, 46, 47]. Environmental surveillance using sewage has already successfully identified enteric pathogens such as polio [48], hepatitis A and E, Adenovirus, and Enterovirus [49]. However, tracking genetic variants of pathogens in wastewater is a complex matrix due to the low content of viral genetic material, the presence of inhibitors (such as humic acid, detergents, phenolic components, and organic wastes), concentration, and the nature of the sample requiring the adaptation of optimization of a method for detection and sequencing of the pathogens like SARS-CoV-2.

To efficiently monitor SARS-CoV-2 variants, methods should remain adaptive and affordable. Due to its cheaper establishment and testing cost, we preferred the Oxford Nanopore MinION platform to recover whole-genome sequences [50] to detect and identify circulatory variants. Indeed, the method we described, using the ONT platform and real-time PCR assays, could provide information within five to seven days of wastewater collection. Moreover, multiple samples as a batch using the ONT platform minimize the cost, as low as $10 [51] per sample (a batch of 96 samples using the LoCost protocol we used). For example, Delta-associated mutations in community wastewater provide strong early evidence that Delta results from individual testing documented lately in Bangladesh. Moreover, this monitoring process is quickly set up and scalable to other contexts matched with Bangladesh’s sanitation system.

The regular diagnostic test for COVID-19 clinical sampling costs approximately $30 per sample [52]. Therefore, covering a large population with a biological sample often becomes a limiting factor in low- and middle-income countries, which hinders establishing a surveillance system for infectious diseases. Wastewater is still cost-efficient for epidemiological monitoring since the sequencing of the SARS-CoV-2 genome; the sample gives information at a population level compared to an individual level. Another significant finding of this study is that the presented method provides a reliable tool for selecting sampling points from a complex sanitation system with a dense population. This is necessary for cost-effectiveness and quick outcome evaluation to represent a large catchment area.

## Limitation

One limitation of the study is that it was conducted only in selected communities. If we could run the analysis in more communities or larger catchment areas, it could be possible to establish an early warning system. Further, out of the 504 samples of wastewater collected for analysis, only a small subset of (*n* = 10) samples was randomly selected for genome sequencing and detailed analysis of genetic mutations in SARS-CoV-2 because only thirty samples had *C*_t_ values ≤ 32.9. Although we could recover good genome coverage, full coverage was not achieved. This could be due to a number of factors, including the low concentration of viral particles and degraded viral RNA in the wastewater. The presence of very low levels of viruses and contaminants hinders full-length genome sequencing.

## Conclusions

We conducted a study on complex sanitation systems that is reproducible in areas of low sanitation coverage worldwide. These findings imply that monitoring wastewater can be a helpful tool for identifying symptomatic and asymptomatic COVID-19 patients in the community. Therefore, early detection through wastewater monitoring can give public health officials supplementary information to rapidly triangulate the prevalence of circulating variants within a community; implementing wastewater surveillance is crucial for current and future outbreaks.

## Supplementary Information

**Additional file 1. Figure S1:** The weekly collection of wastewater samples from the same locations, covering the time frame from September 2020 to May 2021. **Figure S2:** A comparison of the viral load in the sewer network with the number of national COVID-19 cases.

## Data Availability

The complete nucleotide sequences of these SARS-CoV-2 strains have been deposited in the GISAID database under the accession numbers; EPI_ISL_17398164, EPI_ISL_17398165, EPI_ISL_17398166, EPI_ISL_17398167, EPI_ISL_17398168, EPI_ISL_17398169, EPI_ISL_17398170, EPI_ISL_17398171, EPI_ISL_17398172, EPI_ISL_17398173.
