# Supplementary material for: Monitoring SARS-CoV-2 variants in wastewater of Dhaka City, Bangladesh: approach to complement public health surveillance systems
Source: Hum Genomics. 2023 Jul 7;17:58. doi: 10.1186/s40246-023-00505-4 (PMC10326934; doi:10.1186/s40246-023-00505-4)
Supplement: Supplementary file 1 — Additional file 1. Figure S1: The weekly collection of wastewater samples from the same locations, covering the time frame from September 2020 to May 2021. Figure S2: A comparison of the viral load in the sewer network with the number of national COVID-19 cases. [file 40246_2023_505_MOESM1_ESM.pptx]

## Slide 1
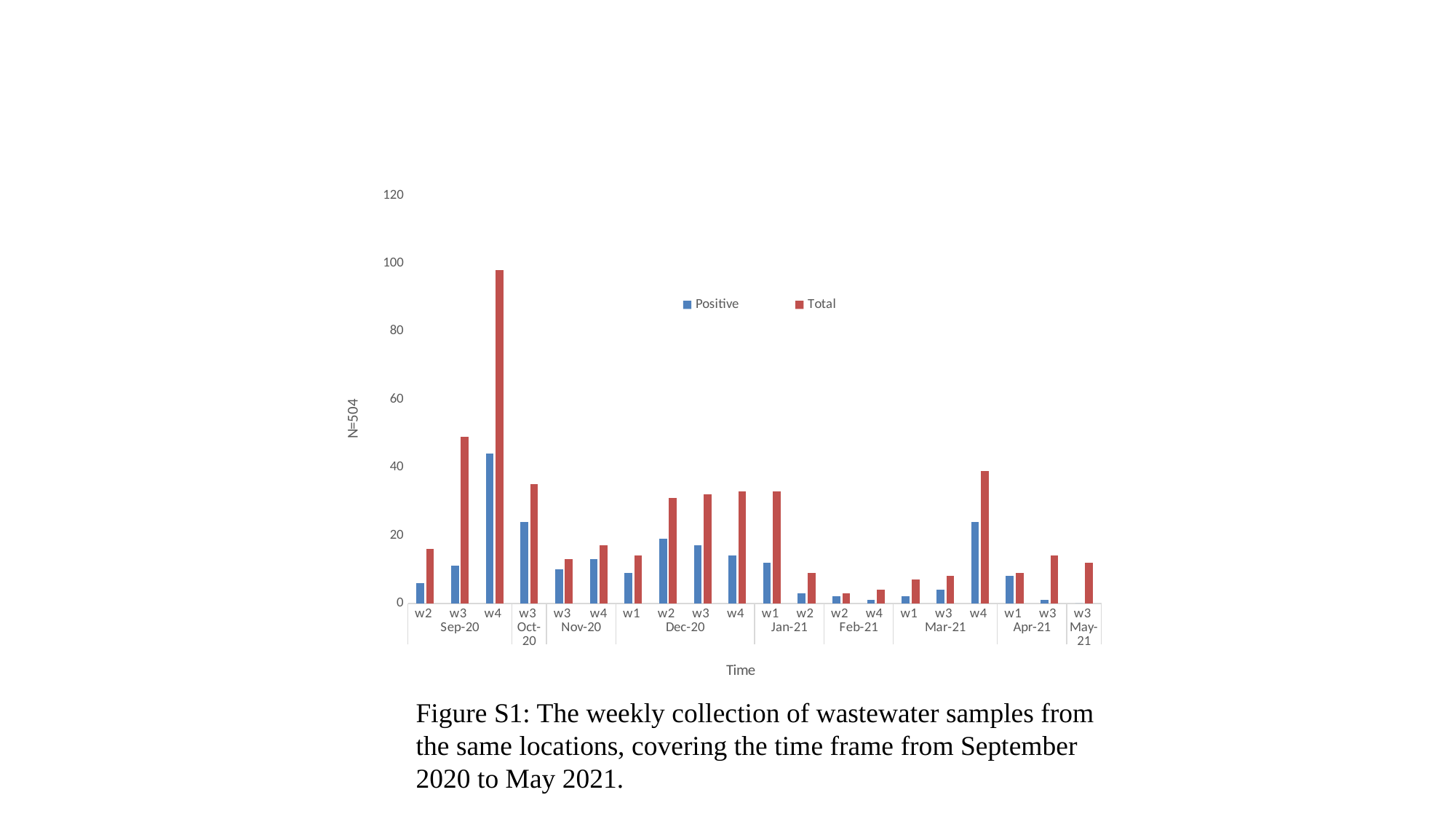

### Chart
| Category | Positive | Total |
|---|---|---|
| w2 | 6.0 | 16.0 |
| w3 | 11.0 | 49.0 |
| w4 | 44.0 | 98.0 |
| w3 | 24.0 | 35.0 |
| w3 | 10.0 | 13.0 |
| w4 | 13.0 | 17.0 |
| w1 | 9.0 | 14.0 |
| w2 | 19.0 | 31.0 |
| w3 | 17.0 | 32.0 |
| w4 | 14.0 | 33.0 |
| w1 | 12.0 | 33.0 |
| w2 | 3.0 | 9.0 |
| w2 | 2.0 | 3.0 |
| w4 | 1.0 | 4.0 |
| w1 | 2.0 | 7.0 |
| w3 | 4.0 | 8.0 |
| w4 | 24.0 | 39.0 |
| w1 | 8.0 | 9.0 |
| w3 | 1.0 | 14.0 |
| w3 | 0.0 | 12.0 |Figure S1: The weekly collection of wastewater samples from the same locations, covering the time frame from September 2020 to May 2021.

## Slide 2
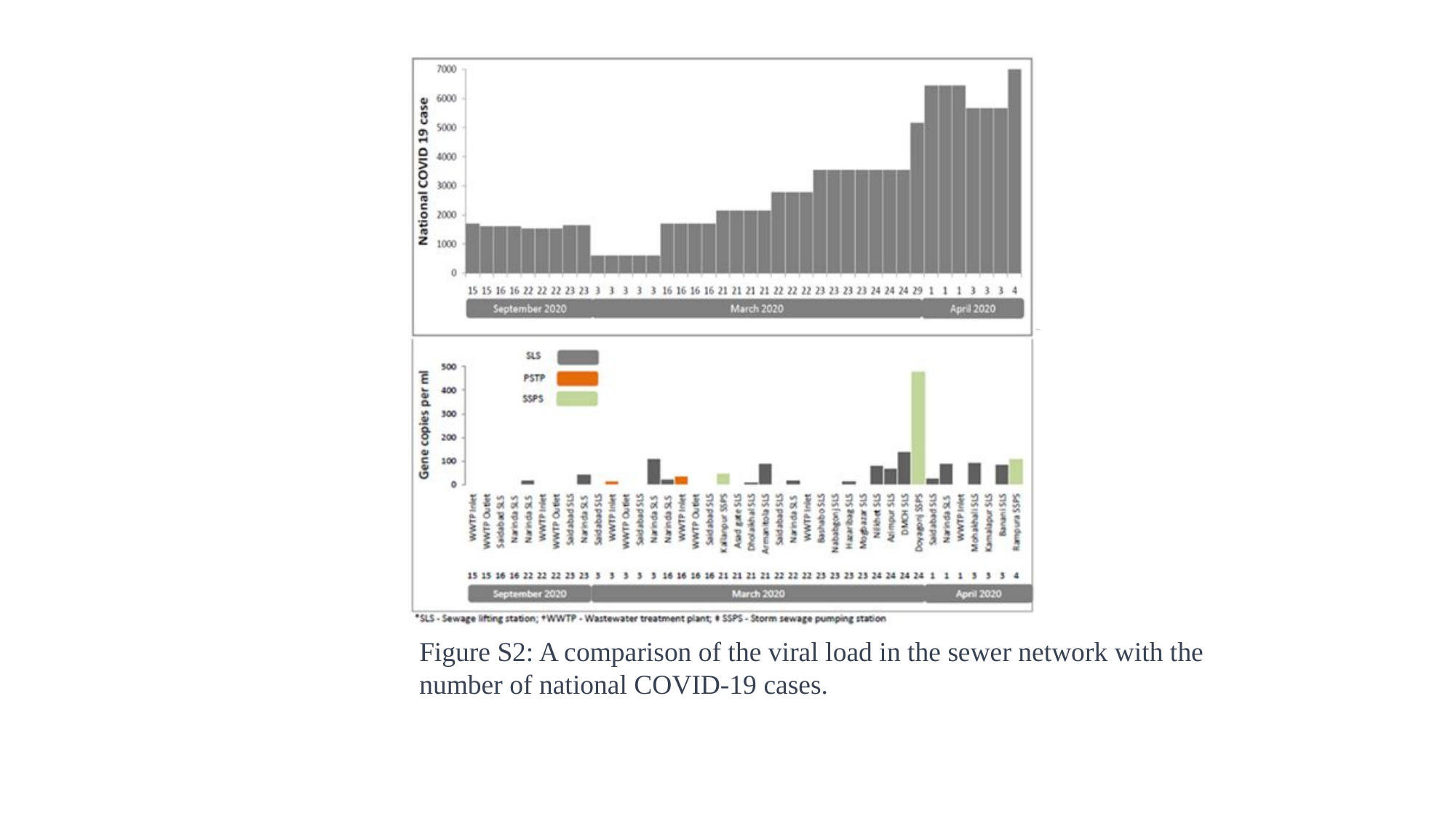

Figure S2: A comparison of the viral load in the sewer network with the number of national COVID-19 cases.
